# Understanding Lifelong Factors and Prediction Models of Social Functioning After Psychosis Onset Using the Large-Scale GROUP Cohort Study

**DOI:** 10.1093/schbul/sbad046

**Published:** 2023-04-27

**Authors:** Natalia Tiles-Sar, Tesfa Dejenie Habtewold, Edith J Liemburg, Lisette van der Meer, Richard Bruggeman, Behrooz Z Alizadeh, GROUP investigators, Richard Bruggeman, Behrooz Z Alizadeh, Therese van Amelsvoort, Agna A Bartels-Velthuis, Lieuwe de Haan, Frederike Schirmbeck, Claudia J P Simons, Jim van Os

**Affiliations:** Department of Epidemiology, University Medical Center Groningen, University of Groningen, Groningen, The Netherlands; Department of Psychiatry, Rob Giel Research Center, University Medical Center Groningen, University Center for Psychiatry, University of Groningen, Groningen, The Netherlands; Department of Epidemiology, University Medical Center Groningen, University of Groningen, Groningen, The Netherlands; Department of Psychiatry, Rob Giel Research Center, University Medical Center Groningen, University Center for Psychiatry, University of Groningen, Groningen, The Netherlands; Department of Clinical and Developmental Neuropsychology, Universityof Groningen, Groningen, The Netherlands; Department of Rehabilitation, Lentis Psychiatric Institute, Zuidlaren, The Netherlands; Department of Psychiatry, Rob Giel Research Center, University Medical Center Groningen, University Center for Psychiatry, University of Groningen, Groningen, The Netherlands; Department of Epidemiology, University Medical Center Groningen, University of Groningen, Groningen, The Netherlands; Department of Psychiatry, University of Groningen, University Medical Center Groningen, University Center for Psychiatry, Rob Giel Research Center, Groningen, The Netherlands; Department of Clinical and Developmental Neuropsychology, University of Groningen, Groningen, The Netherlands; Department of Epidemiology, University Medical Center Groningen, Groningen, The Netherlands; Department of Psychiatry and Neuropsychology, School for Mental Health and Neuroscience, Maastricht University Medical CenterMaastricht, The Netherlands; Department of Psychiatry, University of Groningen, University Medical Center Groningen, University Center for Psychiatry, Rob Giel Research Center, Groningen, The Netherlands; Department of Psychiatry, Amsterdam UMC, University of Amsterdam, Amsterdam, The Netherlands; Research Department, Arkin Institute for Mental Health, Amsterdam, The Netherlands; Department of Psychiatry, Amsterdam UMC, University of Amsterdam, Amsterdam, The Netherlands; Research Department, Arkin Institute for Mental Health, Amsterdam, The Netherlands; Department of Psychiatry and Neuropsychology, School for Mental Health and Neuroscience, Maastricht University Medical CenterMaastricht, The Netherlands; GGzE Institute for Mental Health Care, Eindhoven, The Netherlands; Department of Psychiatry, University Medical Center Utrecht Brain Centre Rudolf Magnus, Utrecht University, Utrecht, The Netherlands; Department of Psychosis Studies, King’s College London, King’s Health Partners, Institute of Psychiatry, London, UK

**Keywords:** schizophrenia, association, mixed-effect model/trajectories, follow-up

## Abstract

**Background and hypothesis:**

Current rates of poor social functioning (SF) in people with psychosis history reach 80% worldwide. We aimed to identify a core set of lifelong predictors and build prediction models of SF after psychosis onset.

**Study design:**

We utilized data of 1119 patients from the Genetic Risk and Outcome in Psychosis (GROUP) longitudinal Dutch cohort. First, we applied group-based trajectory modeling to identify premorbid adjustment trajectories. We further investigated the association between the premorbid adjustment trajectories, six-year-long cognitive deficits, positive, and negative symptoms trajectories, and SF at 3-year and 6-year follow-ups. Next, we checked associations between demographics, clinical, and environmental factors measured at the baseline and SF at follow-up. Finally, we built and internally validated 2 predictive models of SF.

**Study results:**

We found all trajectories were significantly associated with SF (*P* < .01), explaining up to 16% of SF variation (*R*^2^ 0.15 for 3- and 0.16 for 6-year follow-up). Demographics (sex, ethnicity, age, education), clinical parameters (genetic predisposition, illness duration, psychotic episodes, cannabis use), and environment (childhood trauma, number of moves, marriage, employment, urbanicity, unmet needs of social support) were also significantly associated with SF. After validation, final prediction models explained a variance up to 27% (95% CI: 0.23, 0.30) at 3-year and 26% (95% CI: 0.22, 0.31) at 6-year follow-up.

**Conclusions:**

We found a core set of lifelong predictors of SF. Yet, the performance of our prediction models was moderate.

## Introduction

Social functioning (SF) corresponds to a person’s ability to socialize and achieve social goals, for example, being independent, employed, having an education, friends, and romantic relationships, and participating in social activities.^1^ Social functioning is related to social connectedness (i.e., the need to belong and relate), self-esteem, and self-actualization.^2,3^ SF disturbance may lead to anxiety, depression, risk of suicide, increased early mortality, impacts inversely the patients’ quality of life, and lead to a burden for relatives, community, and society in general.^4,5^ Rates of poor SF in people with schizophrenia vary from 82.2% in North Africa and the Middle East to 65% in Northern Europe.^6^ A recent meta-analysis on changes in SF during the course of schizophrenia found a moderate improvement in overall SF, but only minor improvement in vocational functioning, prosocial behavior, activities, and independence.^7^ A systematic review reported annual costs of schizophrenia across 24 countries ranging from US$94 to US$102 million, with 50%–85% of these costs attributed to inadequate SF.^8^

Poor SF may start early in life. It often precedes the clinical onset of psychosis and is an indicator of vulnerability to develop psychosis.^9–11^ Poor SF is linked to worse cognitive functioning, severe negative and, to a lesser extent, positive symptoms.^12–15^ In turn, severe clinical symptoms are associated with a further decline in SF.^16–18^ We and others have shown that people with schizophrenia have a highly heterogeneous disease course as divergent longitudinal trajectories of positive, and negative symptoms, cognitive function, and SF have been reported.^19–21^ Although uninvestigated, it is conceivable that SF differs between the trajectories, subgroups of people with different courses of schizophrenia. Besides, patients’ characteristics (e.g., sex, substance abuse, ethnicity/immigration status, education) and environmental factors (e.g., marriage, familial attitudes, childhood trauma) are also associated with SF.^21–26^ However, the majority of studies on SF report the univariable effect sizes or adjusted only for a few covariates,^25–30^ developed models with a limited number of predictors,^31–33^ conducted path analysis.^23,34^ One study showed that childhood and adolescence experience, disease-related factors, temperament traits, and brain morphology are predictive for SF.^35^ Duration of untreated psychosis was also highlighted.^30^ These scattered findings hamper vision of reasons for high prevalence of poor SF, only moderate improvements in SF, and thus form a barrier for proper management as for the past 50 years.

A prediction model of SF might offer a solution for identifying patients at high risk of poor SF and highlighting the strongest persistent predictors. The predictive model would allow clinicians to adjust the management plan and recommend tailored interventions for SF depending on personal and clinical characteristics. To follow modern theories and evidence, several predictors’ groups should be considered—clinical, non-clinical, and environmental factors. Additionally, consideration of trajectories instead of single scores can offer a meaningful division of patients’ groups, their further comparison and identification of high-risk profiles. Previously, SF at 12 months was predicted with age, negative symptoms, and pretreatment SF in 56 patients at 59% explained variance,^15^ 18 months follow-up was predicted by psychopathology and neurocognition in 49 patients at 25% explained variance,^36^ 4–6 years follow-up was predicted by PANSS total score at baseline and duration of untreated psychosis in 74 patients at 20% explained variance.^16^ Similar outcomes, such as social and vocational recovery at 1 year or poor social outcome at 3- and 5-year follow-up, were predicted with higher accuracy of 50%–90%.^24,37^ The models exhibited medium to high performance, mostly predicted short-term SF commonly 1-year follow-up or shorter, had a small sample size of <200 patients, and did not include trajectories.^15,16,24,36,38^

We aimed to identify a core set of lifelong predictors, such as baseline factors, premorbid adjustment, and clinical trajectories, of SF after psychosis onset and to build prediction models using the strongest associations. We hypothesized that social functioning is predictable by premorbid adjustment trajectories, clinical (i.e., cognitive deficits, positive and negative symptoms) trajectories, and measured at baseline factors (i.e., demographics, disease characteristics, substance abuse, genetic susceptibility, environment). Specifically, we investigated the relationship between premorbid adjustment and clinical trajectories, and, further, their association with SF after psychosis onset. Next, we built and internally validate two prediction models of SF at 3- and 6-year follow-ups using significant trajectories and measured at baseline predictors.

## Methods

### Study Design and Population

This study uses data of the Dutch cohort Genetic Risk and Outcome in Psychosis (GROUP; data release 7.0), a longitudinal study with measurements taken at the baseline (first wave), 3-year (second wave), and 6-year (third wave) follow-up.^39^ The details of the study are published elsewhere.^39^ At baseline, 1119 patients with a psychotic disorder were recruited by 4 Dutch university medical centers. Overall, 744 (66 %) patients completed the assessment at the second wave and 599 (53 %) at the third wave. Majority of patients were adult Caucasian men diagnosed with schizophrenia who had average onset at 23 years and average illness duration of 5 years (further details reported in the Results).

### Outcome

SF was measured with the Social Functioning Scale (SFS) at 3- and 6-year follow-up. The SFS is a standard measure that was developed and validated for people with schizophrenia by Birchwood et al.^40^ SFS includes the following subscales: social engagement or withdrawal, interpersonal functioning, current social activities, recreational activities, independence-competence, independence-performance, and employment.^40^ The reliability of the SFS measured by Cronbach’s alpha in our data was 0.80 for the second and 0.79 for the third wave. The total SFS score was generated by taking a mean of subscales’ scores, a higher score meaning better SF.

### Predictors

#### Premorbid Adjustment Trajectories.

The researchers scored the Premorbid Adjustment Scale (PAS) based on gathered information from patients’ parents (otherwise, from siblings). PAS is a retrospective measure (i.e., administered at baseline) that reflects social life and school performance in three life periods: childhood (<12 years), early adolescence (12–16 years), and late adolescence (16–19 years).^41^ The scoring range of each item is 0–6. Higher scores indicate worse premorbid adjustment. Premorbid adjustment trajectories are subgroups that were identified using group-based trajectory modeling (see statistical analysis) based on the average PAS scores at each period.^41,42^

#### Cognitive Deficits, Positive, and Negative Symptoms Trajectories.

Five cognitive deficits trajectories based on composite cognitive score measures over a 6-year follow-up have earlier been distinguished by our research group using group-based trajectory modeling.^43^ The trajectories showed relatively stable functioning over time: high (10.1%) and normal (31.5%) cognition, mild (41.6%), moderate (14.4%), or severe (2.3%) deficit. To increase power and balance the subgroups, we merged the trajectories based on the severity level into “high to normal cognition” (41.6%), “mild cognitive deficit” (41.6%), and “moderate to severe cognitive deficit” (16.7%). Positive and negative symptom trajectories have been previously modeled based on the relevant domains of the Positive and Negative Syndrome Scale (PANSS).^44^ We distinguished three subgroups that demonstrated relatively stable trajectories of positive symptoms, where 70.4% of patients showed “low” levels of positive symptoms, 21.2% “moderate” and 8.4% “severe” levels. Also, we have identified three negative symptoms trajectories where 74.0% of patients showed “low”, 14.3% “high, decreasing severity”, and 11.7% “high, increasing severity” of negative symptoms over 6 years of follow-up.^44^

#### Measured at Baseline Patients’ Characteristics and Environmental Factors.

In addition to premorbid adjustment, cognitive and symptoms trajectories, we included 21 predictors (Supplementary Table S1), such as age (in years), sex (male/female), ethnicity (Caucasians/no Caucasians), education (highest achieved education, by Verhage),^45^ psychotic episodes (count), age of psychosis onset (in years), duration of illness (in years), antipsychotic use (chlorpromazine equivalent), alcohol (consumed units in a week), cannabis (“none”, “less than weekly”, “weekly”, “daily”), polygenic risk score (change of 1 standard deviation), number of moves before admission (count), current urbanicity (“not urban”, “little to strong urban”, “very strongly urban”), urbanicity at birth (“not urban”, “little to strong urban”, “very strongly urban”), living conditions (“single”, “with parent(s)”, “with a partner/family”, “sheltered living”), employment (“none”, “full-time”, “part-time”), marital status (“not married”, “married/living together”, “divorced”), parents loss (yes/no), having children (yes/no), unmet needs of social support (Camberwell Assessment of Need scale)^46^ and childhood trauma (Childhood Trauma Questionnaire).^47^ Predictors were chosen a priory based on extensive literature review, GROUP investigators’ opinions, potential predictors’ relevance to SF, and availability in the GROUP dataset. All predictors were measured at baseline, except for the childhood trauma questionnaire, which was administered at baseline (Maastricht) or the second wave (the other research sites).

### Data Analysis

#### Missing Values and Drop-out.

 A full set of observations was available for the family ID (as some patients came from the same family and were grouped accordingly), medical center, sex, and identified trajectories (missing values in the original scales were handled with maximum likelihood). Eight predictors had <5% missing values, 7 variables had 5%–20% missing values, and 5 variables had 20%–40% missing values (Supplementary Table S2). The SFS also contained missingness, 33.5% and 46.5% at the second and third waves, respectively. The missingness was either related to drop-out or the information was not collected. After confirming MAR assumption, we implemented a Bayesian Stochastic regression single imputation that resulted in 1045 complete cases (Supplementary Methods).^48^

#### Power Calculation.

See in detail described in Supplementary Methods.

#### Statistical Modeling.

 Preceding the statistical modeling, we compared SFS values at the two waves by paired *t*-test and paired samples correlation.

*Group-based Trajectory Modeling* (GBTM) was used to determine the best-fitting classification model of premorbid adjustment trajectories (see details in Supplementary Methods).^42,49^ The same method was applied earlier to distinguish cognitive deficits, positive, and negative symptoms trajectories.^43,44^ The data analysis was implemented in Stata/SE 14.2.^50^

*Trajectories Analysis* included independence analysis with Cramer’s *V* (<.05 no or very weak, 0.05–0.10 weak, 0.10–0.15 moderate, 0.15–0.25 strong, and >0.25 very strong association).^51^ Further, we examined relationships between premorbid adjustment, cognitive, and symptom trajectories and the SFS at wave 2 and wave 3 in univariable analyses and estimated the unique contribution of every trajectory in the multivariable model.

**Figure 1.**
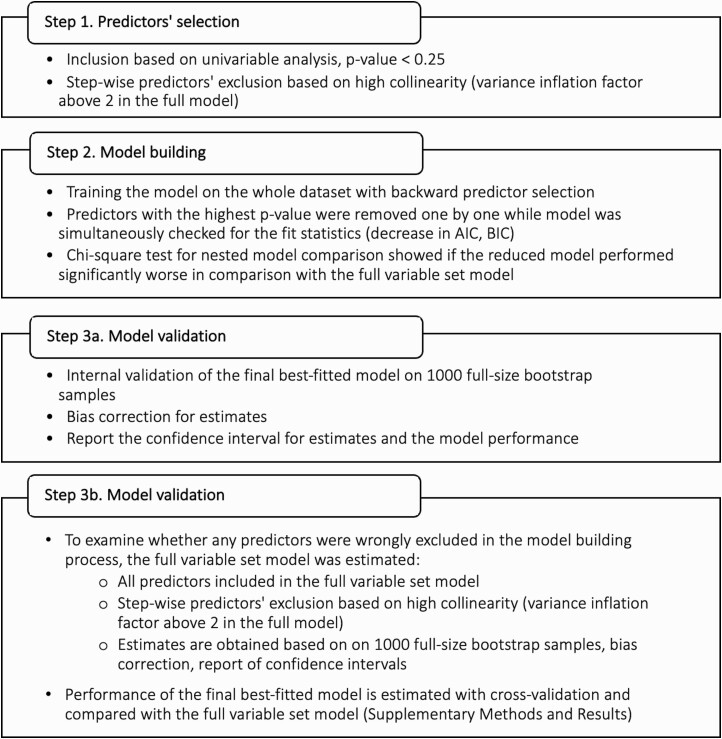
Process of the model development.

*At the stage of model building*, we estimated effect sizes of baseline predictors in the univariable analysis. Next, we built and internally validated the prediction models utilizing a 2-step validation procedure (Figure 1 and Supplementary Methods).^52^ The results were reported in agreement with the TRIPOD statement and the proposed checklist (Supplementary Table S3).^53^

*Linear Mixed-Effect Model* was used to estimate the predictive power of selected predictors on SF and further develop the prediction model. We treated predictors of interest as a fixed effect, and family ID (in total, 52 families had 2–4 patients) nested within 4 clinical centers as a two-level random effect. The analysis was conducted using the lme4 package in R.^54^ For each predictor unstandardized regression coefficients have been reported. *P*-values for fixed effects were obtained via Satterthwaite’s degrees of freedom method available in the lmerTest package.^55^ To compare model fit of nested models we used AIC, BIC, log-likelihood, and Chi-square test. We reported marginal (the variance explained by the fixed effects only) and conditional (the variance explained by the fixed and random effects together) pseudo-R-squared for Mixed-Effect models using MuMIn package.^56^

## Results

### Descriptive of the Sample

In total, 1119 patients were included (283 patients from AMC, Amsterdam, 287 from UMCG Groningen, 306 from MUMC Maastricht, and 243 from UMCU Utrecht). Schizophrenia was the most common diagnosis (65.1% followed by schizoaffective disorder in 10.8% and unspecified psychosis in 10.6% of patients). Patients (mean age 27.59 ± [SD]7.97) were mostly male (76.10%) and Caucasian (79.40%). Among participants, 54.7% participants graduated from high school or had a higher education level. Patients’ illness duration was, on average, 4.53 ± 4.48 years (with a mean age of onset at 23.06 ± 7.80) and they experienced 1.73 ± 1.61 psychotic episodes at the baseline, while 41.9% of the patients had an onset of psychosis in the past 2 years. Almost half of the patients (43.10%) were consuming cannabis daily, patients used 6.54 ± 12.03 units of alcohol per week. Many patients were living alone (35.00%) or with parent(s) (42.60%), while 11.40% were living with a partner/family and 10.90% in sheltered housing. Most patients were not married (86.30%) and 46.30% did not have a job at the baseline (Supplementary Table S2).

In the observed data, SFS measures at both waves were normally distributed with a mean of 112.51 ± 9.36 at the second wave and 113.91 ± 9.00 at the third wave, yielding a significant (*p* < .001) mean difference of −0.96 ± 6.71 and a correlation coefficient of 0.74 between the two SFS measures (Supplementary Table S4). In the imputed data, SFS scores were comparable, being 112.26 ± 9.47 at the second and 113.23 ± 9.11 at the third wave with a correlation of 0.67 between the two measures and a mean difference of −0.97 ± 7.56.

### Premorbid Adjustment Trajectories

**Figure 2.**
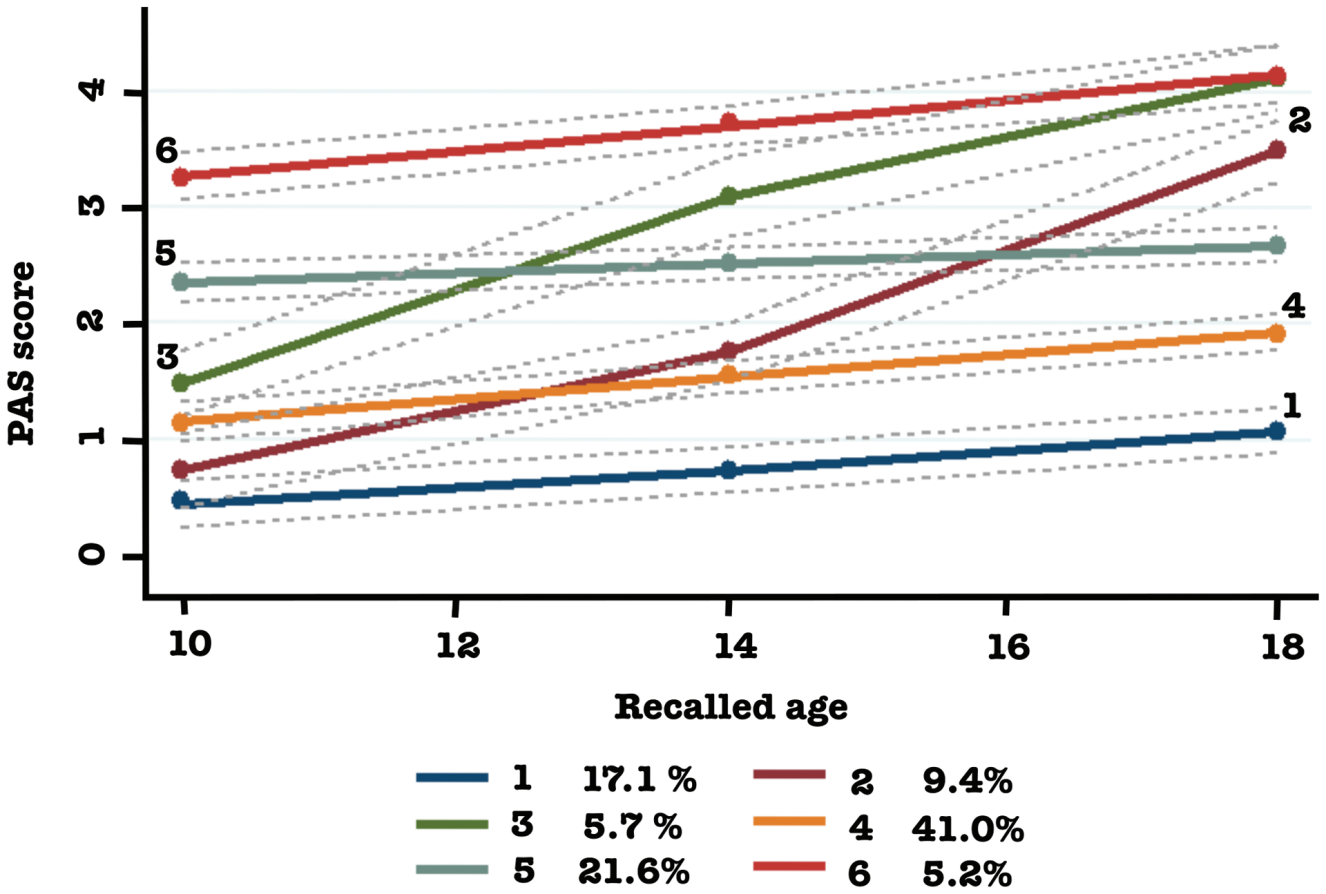
Premorbid adjustment trajectories. Recalled age is the approximated age of participants’ recalled premorbid adjustment; the lines represent the following trajectories of impairment: (1) “normal, slow decrease,” (2) “normal, rapid decrease,” (3) “mild, rapid decrease,” (4) “mild, slow decrease,” (5) “moderate, slow decrease,” (6) “severe, slow decrease.”

The PAS was filled in for all periods in 910 patients. The mean PAS scores were for 1.38 ± 0.95 for childhood, 1.84 ± 0.95 for early adolescence, and 2.31 ± 1.09 for late adolescence (all pairs differed significantly at *P*-value <.001 by paired samples *t*-test). Using GBTM, we distinguished 6 subgroups (Supplementary Results and Tables S5 and S6). Identified subgroups vary in initial severity level and further form and speed of decline (Figure 2). Group 5 had relatively stable PAS over time, groups 2 and 3 had a parabola shape and the biggest PAS deterioration, while the other groups had a modest decline in PAS. To reduce degrees of freedom in the subsequent analyses and given the observed frequencies and the specification of trajectories, trajectories 1 and 4 were combined into the group “normal to mild, slow decrease” (65.8% of the patients), trajectories 2 and 3 were combined into “normal to mild, rapid decrease” (11.2%), and trajectories 5 and 6 were merged into “moderate to severe, slow decrease” (23.1%).

### Premorbid Adjustment, Cognitive Deficits, Symptoms Trajectories, and SF

The frequencies of premorbid adjustment and clinical trajectories are reported in Supplementary Table S2. We observed moderate significant (*P* value <.001) correlations between trajectories of premorbid adjustment and cognitive deficits (Cramer’s *V* value = 0.104), premorbid adjustment and positive symptoms (0.104), premorbid adjustment and negative symptoms (0.129), cognitive deficit and negative symptoms (0.129), cognitive deficit and positive symptoms (0.103). Strong correlation was observed between positive and negative symptoms trajectories (0.193). In other words, patients’ course of the premorbid adjustment, cognitive deficits, and symptoms is not independent.

Results of association analyses are presented in Table 1 and Supplementary Table S7. All trajectories were significantly associated with SF in both waves of assessment. Thus, in comparison to the best functioning (i.e., reference) trajectory subgroup, more severe trajectories had significantly worse SF. The trajectories’ effect widely varied between per each domain and trajectory form. For example, subgroup with a high level of negative symptoms with increasing severity had, on average, 5 points lower SF than group with low negative symptoms. Group who had high negative symptoms but decreasing severity showed 3 points lower SF at 3-year follow-up and 1.5 point lower at 6-year follow-up. The explained variance (conditional *R*^2^/marginal *R*^2^) of the multivariable models was 0.30/0.15 for the 3- and 0.44/0.16 for the 6-year follow-up. In comparison to the null model (no fixed effect is included), the models with trajectories performed significantly better (fit characteristics reported in Supplementary Results).

**Table 1.** Unstandardized estimates of relationship between premorbid adjustment, cognitive deficits, symptoms trajectories and SF^a^ at 3- and 6-year follow-up.

Parameter	3-Year follow-up		6-Year follow-up	
	Univariable^b^	Multivariable^c^	Univariable^b^	Multivariable^c^
	Estimate (SE)	Estimate (SE)	Estimate (SE)	Estimate (SE)
*R* ^2^ (marginal)		0.15		0.16
Intercept		117.34 (0.81)		118.59 (0.67)
Premorbid adjustment trajectories				
Normal to mild, slow decrease	Ref.	Ref.	Ref.	Ref.
Normal to mild, rapid decrease	−2.04 (0.75)^*^	−1.23 (0.70)	−2.33 (0.72)^**^	−1.52 (0.67)^*^
Moderate to severe, slow decrease	−4.95 (0.88)^**^	−2.78 (0.84)^**^	−5.25 (0.84)^**^	−3.09 (0.80)^**^
Cognitive deficits trajectories				
High to normal cognition	Ref.	Ref.	Ref.	Ref.
Mild cognitive deficit	−3.07 (0.60)^**^	−2.29 (0.58)^**^	−3.46 (0.58)^**^	−2.71 (0.55)^**^
Moderate to severe cognitive deficit	−5.84 (0.80)^**^	−3.94 (0.77)^**^	−6.11 (0.76)^**^	−4.34 (0.74)^**^
Negative symptoms trajectories				
Low	Ref.	Ref.	Ref.	Ref.
High, decreasing severity	−4.63 (0.79)^**^	−3.13 (0.78)^**^	−3.22 (0.76)^**^	−1.49 (0.74)^*^
High, increasing severity	−7.36 (0.85)^**^	−5.24 (0.86)^**^	−6.99 (0.83)^**^	−4.62 (0.82)^**^
Positive symptoms trajectories				
Low	Ref.	Ref.	Ref.	Ref.
Moderate	−5.08 (0.68)^**^	−3.56 (0.66)^**^	−4.84 (0.65)^**^	−3.48 (0.64)^**^
Severe	−5.25 (0.99)^**^	−2.91 (0.98)^*^	−6.25 (0.95)^**^	−4.21 (0.94)^**^

*Note*: The presented values show average difference in SF (outcome) between each trajectory (subgroup) compared to the Ref. trajectory (subgroup). *R*^2^ (marginal) represents the variance explained by the fixed effects only; SE, standart error; Ref., reference group.

^a^Social functioning (SF): numeric outcome, higher score represents better social functioning.

^b^Univariable analysis, SF was regressed only over the corresponding trajectory domain (e.g., only premorbid adjustment).

^c^Multivariable models includes trajectories of premorbid adjustment, cognitive deficite, negative symtoms, and positive symtoms.

^*^ *P* value <.05.

^**^ *P* value <.001.

### Prediction Models of SF

In the full variable set model, living conditions and age of onset were excluded due to high collinearity. Based on the univariable analysis and collinearity assessment, age of onset, antipsychotic use, living conditions, and alcohol use were excluded from further model development. Further, based on backward predictor selection procedure, urbanicity at birth, loss of parents, and having children were excluded from the final best-fitted model as they were not associated with SF at *p* < .05 (Supplementary Table S8). The explained variance (conditional *R*^2^/marginal *R*^2^) of the final best-fitted models was 0.30/0.28 for the 3- and 0.63/0.28 for the 6-year follow-up (details reported in Supplementary Results, page 3). Therefore, the random effect accounted for more than half of the explained variance for a 6-year follow-up. The bootstrap estimates obtained from the final best-fitted model and full set variable model are presented in Table 2. Overall, obtained values were similar confirming validity of model building process and obtained results. Comparing both models, only alcohol ­additionally showed a significant association for 6-year follow-up with a relatively small effect size.

**Table 2.** Unstandardized estimates of the predictors in the final best-fitted and in the full variable set prediction models of SF^a^ at 3- and 6-year follow-up.

	3-Year follow-up		6-Year follow-up	
	Final best-fitted model	Full variable set model	Final best-fitted model	Full variable set model
Parameter	Estimate [95%CI]	Estimate [95%CI]	Estimate [95%CI]	Estimate [95%CI]
*R* ^2^(marginal)	0.27 [0.23, 0.30]	0.27 [0.23, 0.31]	0.26 [0.22, 0.31]	0.26 [0.22, 0.31]
Intercept	115.52 [112.62, 118.42]	115.58 [112.60, 118.96]	122.54 [119.79, 125.28]	120.60 [117.33, 123.60]
Premorbid adjustment trajectories				
Normal to mild, slow decrease	Ref.	Ref.	Ref.	Ref.
Normal to mild, rapid decrease	−0.74 [−2.03, 0.55]	−0.66 [−1.99, 0.56]	−1.66 [−2.95, −0.37]^*^	−1.65 [−2.95, −0.42]^*^
Moderate to severe, slow decrease	−2.05 [−3.60, −0.50]^*^	−1.70 [−3.41, −0.22]^*^	−3.19 [−4.70, −1.68]^*^	−3.21 [−4.83, −1.80]^*^
Cognitive deficits trajectories				
High to normal cognition	Excluded at previous step (high *p* value)	Ref. −0.78 [−1.78, 0.33] −1.34 [−2.78, 0.07]	Ref.	Ref.
Mild cognitive deficit			−1.93 [−2.97, −0.89]^*^	−1.76 [−2.82, −0.61]^*^
Moderate to severe cognitive deficit			−2.90 [−4.29, −1.51]^*^	−2.61 [−4.06, −1.12]^*^
Negative symptoms trajectories				
Low	Ref.	Ref.	Ref.	Ref.
High, decreasing severity	−2.45 [−3.92, −0.98]^*^	−2.25 [−3.72, −0.79]^*^	−1.02 [−2.43, 0.39]	−0.96 [−2.30, 0.48]
High, increasing severity	−4.13 [−5.68, −2.58]^*^	−4.07 [−5.50, −2.40]^*^	−3.72 [−5.27, −2.17]^*^	−3.47 [−4.98, −1.78]^*^
Positive symptoms trajectories				
Low	Ref.	Ref.	Ref.	Ref.
Moderate	−2.56 [−3.79, −1.33]^*^	−2.33 [−3.53, −1.08]^*^	−2.55 [−3.72, −1.37]^*^	−3.61 [−3.78, −1.38]^*^
Severe	−0.72 [−2.54, 1.10]	−0.77 [−2.81, 1.09]	−1.77 [−3.59, 0.05]	−1.75 [−3.54, −0.01]^*^
Demographical characteristics				
Age at the baseline	−0.17 [−0.25, −0.09]*	−0.17 [−0.26, −0.07]^*^	−0.15 [−0.23, −0.07]^*^	−0.16 [−0.25, −0.07]^*^
Sex: female	2.81 [1.65, 3.97]*	2.76 [1.61, 4.15]^*^	3.03 [1.80, 4.26]^*^	2.95 [1.79, 4.22]^*^
Ethnicity: non-Caucasian	−2.40 [−3.87, −0.93]*	−2.01 [−3.53, −0.74]^*^	−2.73 [−3.95, −1.51]^*^	−2.43 [−3.98, −0.91]^*^
Education at the baseline	0.82 [0.55, 1.10]^*^	0.73 [0.48, 1.04]^*^	Excluded at previous step (high *P*-value)	0.18 [−0.08, 0.44]
Disease characteristics—status at the baseline				
Number of psychotic episodes	Excluded at previous step (high *P*-value)	−0.14 [−0.61, 0.28]	−0.85 [−1.28, −0.42]^*^	−0.81 [−1.23, −0.35]^*^
Duration of illness	−0.11 [−0.23, 0.01]	−0.11 [−0.24, 0.02]	−0.21[−0.33, −0.09]^*^	−0.21 [−0.32, −0.08]^*^
Substance abuse— status at the baseline				
Alcohol	Excluded at previous step (high *P*-value)	0.01 [−0.04, 0.05]	Excluded at previous step (high *P*-value)	0.06 [0.02, 0.11]^*^
Cannabis usage				
None	Ref.	Ref.	Ref.	Ref.
Less than weekly	Ref.	1.07 [−0.81, 3.08]	1.10 [−0.76, 2.96]	0.95 [−0.98, 2.78]
Weekly	2.23 [0.60, 3.86]*	2.71 [0.86, 4.47]^*^	0.97 [−0.74, 2.68]	0.80 [−0.94, 2.59]
Daily	Ref.	0.19 [−0.95, 1.43]	−1.23 [−2.37, −0.09]^*^	−1.47 [−2.68, −0.42]^*^
Genetic susceptibility				
Polygenic risk score for schizophrenia	−0.15 [−0.23, −0.07]*	−0.14 [−0.22, −0.06]^*^	Excluded at previous step (high *P*-value)	−0.02 [−0.09,0.06]
Environmental factors—status at the baseline				
Current urbanicity				
Not urban	Ref.	Ref.	Ref.	Ref.
Little to strong urban	2.56 [1.30, 3.81]^*^	2.47 [1.10, 3.90]^*^	1.22 [−0.01, 2.46]	0.83 [−0.56, 2.23]
Very strongly urban	2.26 [0.91, 3.61]^*^	2.29 [0.79, 3.81]^*^	1.73 [0.40, 3.06]*	1.18 [−0.31, 2.67]
Employment				
None	Ref.	Ref.	Ref.	Ref.
Full-time	2.53 [1.35, 3.71]^*^	2.61 [1.34, 3.77]^*^	2.60 [1.44, 3.76]*	2.31 [1.19, 3.42]^*^
Part-time	1.93 [0.66, 3.21]^*^	2.04 [0.80, 3.27]^*^	0.78 [−0.42, 1.97]	0.68 [−0.48, 1.88]
Marital status				
Not married	Ref.	Ref.	Ref.	Ref.
Married/living together	3.48 [1.58, 5.38]^*^	3.09 [1.20, 5.07]^*^	2.88 [1.08, 4.68]*	3.12 [1.30, 5.06]^*^
Divorced	1.49 [−1.47, 4.45]	1.04 [−1.95, 4.13]	2.47 [−0.30, 5.23]	2.93 [0.01, 5.90]^*^
Unmet needs of social support	−0.22 [−0.40, −0.04]*	−0.23 [−0.42, −0.05]*	−0.39 [−0.56, −0.21]^*^	−0.40 [−0.58, −0.20]^*^
Environmental factors—childhood and adolescence				
Childhood trauma	−1.51 [−2.55, −0.47]^*^	−1.65 [−2.74, −0.60]^*^	Excluded at previous step (high *P*-value)	0.55 [−0.46, 1.52]
Medication use	Excluded at previous step (high *P*-value)	0.33 [−0.19, 0.81]	Excluded at previous step (high *P*-value)	−0.12 [−0.65, 0.34]
Number of moves before the admission	Excluded at previous step (high *P*-value)	0.14 [−0.10, 0.34]	0.20 [0.00, 0.39]	0.19 [−0.05, 0.38]
Urbanicity at birth				
Not urban	Excluded based on backward predictors selection	Ref.	Excluded based on backward predictors selection	Ref.
Little to strong urban		0.14 [−1.00, 1.37]		0.97 [−0.17, 2.23]
Very strongly urban		−1.12 [−2.45, 0.33]		1.02 [−0.45, 2.41]
Lost parent: yes	Excluded based on backward predictors selection	−0.30 [−1.76, 1.25]	Excluded based on backward predictors selection	−0.47 [−2.04, 0.99]
Having children: yes	Excluded based on backward predictors selection	1.29 [−0.21, 2.77]	Excluded based on backward predictors selection	0.03 [−1.51, 1.36]

*Note*: Unstandardized estimates were corrected for bias based on bootstrap samples. *R*^2^ (marginal) represents the variance explained by the fixed effects only; Ref., reference group within categorical variables.

^a^Social functioning (SF): numeric outcome, higher score represents better social functioning.

^*^Significance was decided if the CI contained zero.

**Figure 3.**
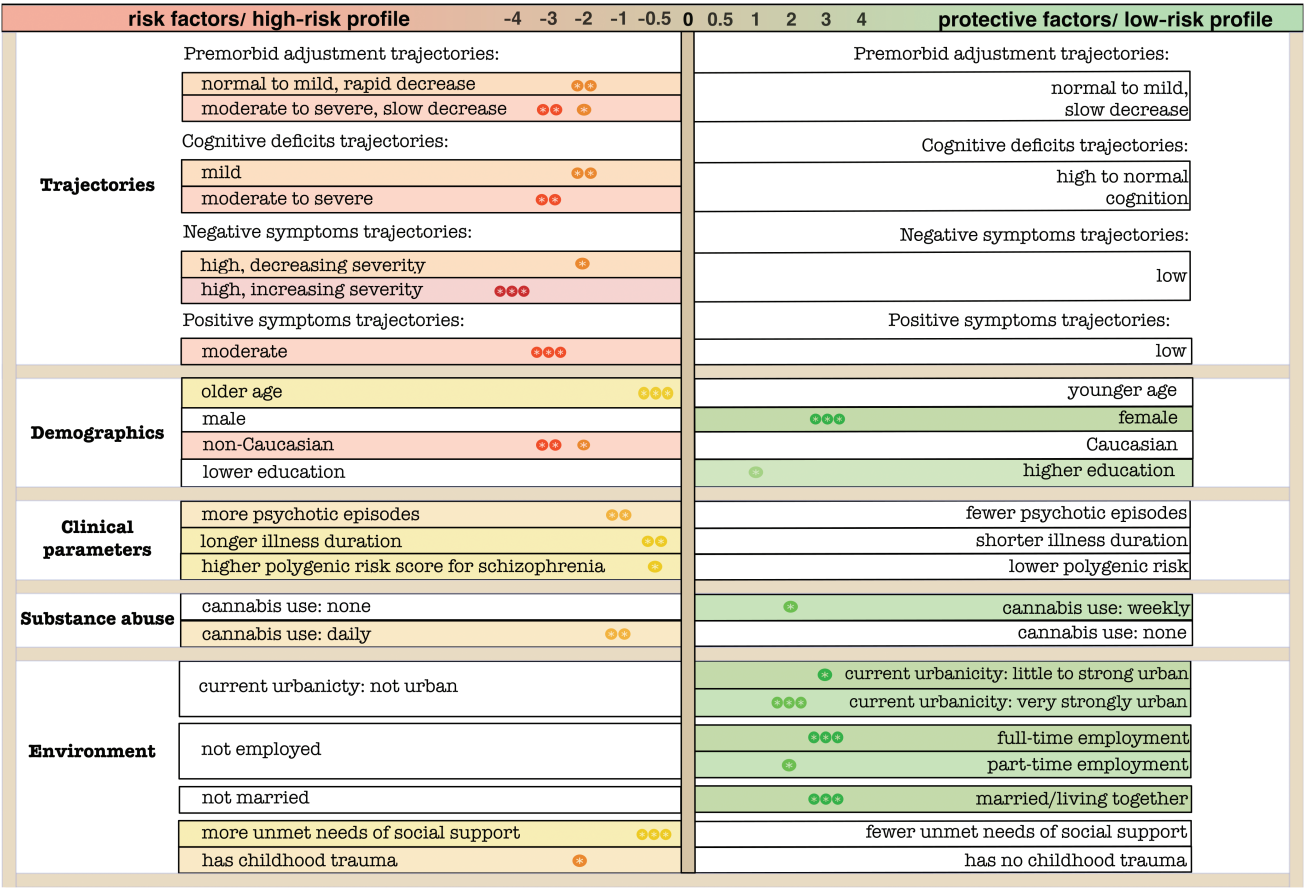
Visualization of (rounded) average estimates of unstandardized regression coefficients for significant predictors (based on bootstrap validation) of SF. Each factor with effect size is placed in colored rectangles, while reference group (for categorical variables) is placed in transcendent rectangles. More intense colors represent bigger effect size. * predictive only for 3-year follow-up. ** predictive only for 6-year follow-up. *** predictive for both waves.

We summarized the validated factors in Figure 3. The left side of the figure shows the list of risk factors that can be combined to identify a high-risk profile, while the right side shows the list of the protective factors that in combination represent a low-risk profile. Thus, belonging to trajectories with more severe symptoms is a risk factor, while having continuously low symptoms is a protective factor for SF at follow-up.

## Discussion

**Figure 4.**
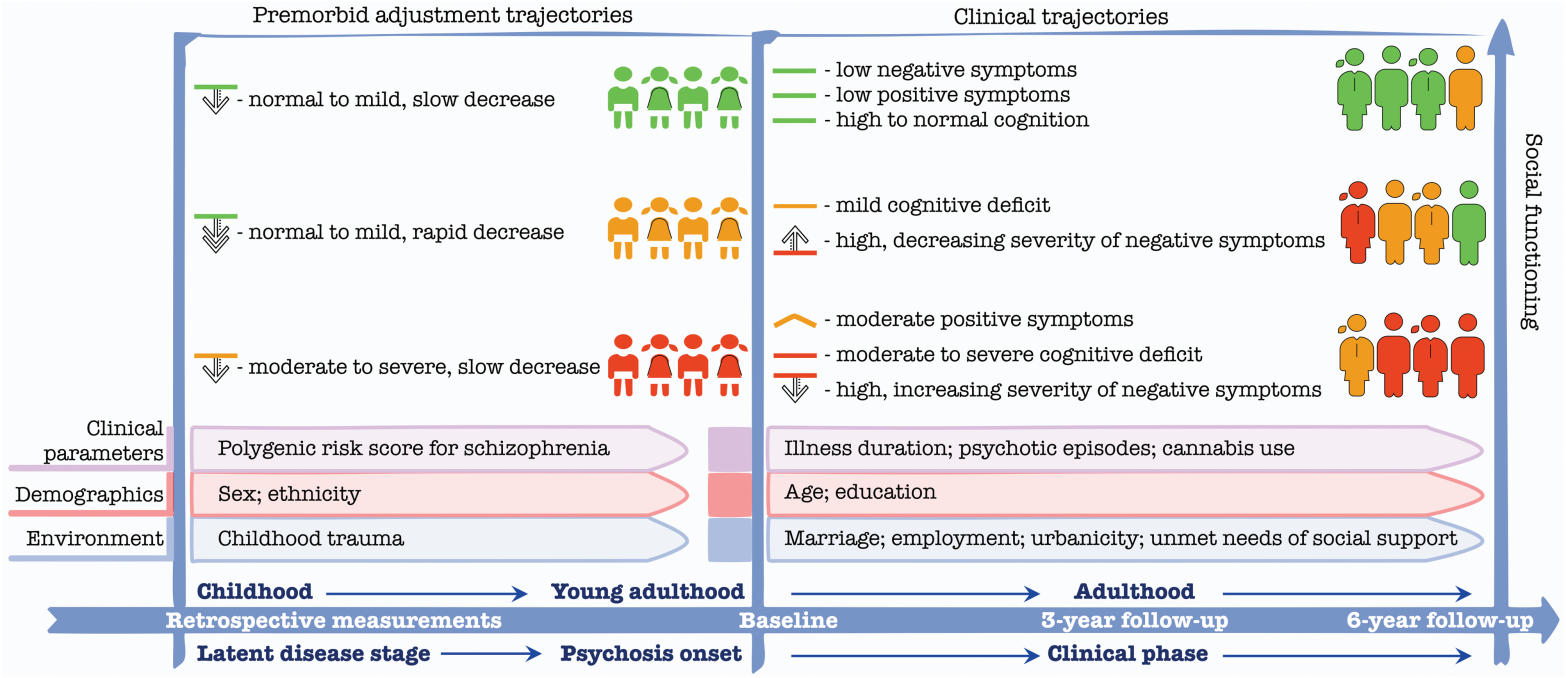
Illustration of the lifelong (significant for either or both waves) predictors of SF. The *X*-axis represents time. The *Y* axis represents social functioning. For illustrative purposes, patients’ subgroups of different premorbid adjustment trajectories are displayed as red (the worst premorbid adjustment trajectory), orange (medium premorbid adjustment impairment), and green (the mildest premorbid adjustment impairment/best functioning group). The individuals within these subgroups further followed clinical trajectories and are shown as the icons on the right side. As a further division of clinical trajectories is not independent, new subgroups are formed highly similar in respect to severity which is shown with color. Next to each trajectory, severity level is shown with horizontal lines and corresponding color. Improvement or decline of symptoms over time is shown with an arrow pointing up or down accordingly.

We aimed to identify a core set of lifelong predictors of SF after psychosis onset and to build prediction models. The main findings are summarized in Figure 4. Our results show that impairment of SF starts early in life and is further associated with the course of cognitive functioning, positive and negative symptoms. Alongside baseline clinical parameters, demographics, and environmental factors improved overall prediction performance.

We found six premorbid adjustment trajectories that varied in their shape but all showed a decline with aging. Previous studies could only distinguish three trajectories (i.e., stable-poor, stable-good, deteriorating).^20,57^ Interestingly, we found a strong effect of the premorbid adjustment trajectories on SF after psychosis onset. Similarly, a previous prospective study showed four relatively stable SF trajectories (preserved, moderately impaired, severely impaired, and profoundly impaired) over a 20-year follow-up with evident differentiation before the disease onset.^9^ We also observed a strong significant effect of clinical trajectories on SF. One study found three cognitive clusters that were associated with SF at baseline and 6-month follow-up.^58^ We uniquely identified that courses of premorbid adjustment, cognition, and symptoms are not independent suggesting a possibility of common underlying mechanism (e.g., genetical, environmental, and/or psychological) across trajectories. To our knowledge, no study has investigated the combined effect of premorbid adjustment and clinical trajectories with SF long after psychosis onset. Our multivariable model suggests that a person from the best functioning trajectories would, on average, have a better (14–17 points higher) SF in the long term than a person from the low functioning trajectories. This corresponds to about 1.5–2.0 standard deviations difference.

We identified other clinical, demographic, and environmental characteristics that predicted SF. Majority of included factors were chosen based on previous studies, and we confirmed predictive performance of age, sex, employment, marriage over 6 years, childhood trauma and education only for 3-year SF, illness duration, and number of psychotic episodes only for 6-year SF. Polygenic risk score for schizophrenia was added based on the assumption that genetic risk for schizophrenia might have a direct relation with SF which was confirmed only for 3-year follow-up (contribution to explaining conditional *R*^2^ was 1%). Cannabis use, although significantly predictive, yielded contradictory results just as existing literature.^59–61^ Ethnicity, social support, and urbanicity were strongly predictive across 6 years although they were mentioned the least across the literature. One study on ethnicity found that “African American subjects had a significantly slower rate of improvement (compared with white subjects) in social functioning”, and another included ethnicity to predict vocational recovery.^24,62^ The difference in SF between Caucasian and non-Caucasians, could be attributed to difference in culture, language barriers, stigma, or ethnic genetic profile differences. Previously strong urbanicity has been linked to a higher incidence of psychosis, although the exact mechanism stays unknown.^63^ Our study shows that living in the rural area is a potential risk factor with a negative impact on SF. The result might be related to fewer options for receiving support and socializing outside of big cities. Another often overlooked factor, social support, was found to be protective for hospitalization and SF.^64^ Having high number of unmet needs can be a burden for patients and lead to prolong recovery. More studies are needed to be validated and understand these results.

While previous prediction models explained 20%–90% in SF, our model performance was moderate and reached maximally 30% for fixed effect only for both follow-ups. The moderate performance of our model can be attributed to longer follow-up; adjustment for random effect; and using trajectories instead of (sub)scale scores. However, familial factor (i.e., being from the same family) accounted for an additional 30% in the prediction of SF at 6-year follow-up, which might be due to the long-term effect (positive or negative) of (intrafamilial) environment evident in the later stage of the disease. Our findings match the results of a recent study which essentially underlines the importance of family support in the recovery process.^65^

### Clinical and Research Implications in Precision Psychiatry

Understanding the heterogeneity of patients is a vital step in designing effective personalized rehabilitation. We showed that patients may follow different premorbid adjustments and clinical trajectories that are associated with long-term SF. Premorbid adjustment and cognitive trajectories (as they remained constant during follow-up) can be assessed at the intake, while negative and positive symptoms might require longer follow-up. Other factors were also important in distinguishing high-risk profile group, characterized as unmarried older non-educated non-Caucasian men living in a rural area with multiple unmet needs. We included the example of the prognosis tool based on our findings in Supplementary Table S9.

### Facilitation of Social Functioning in Practice

Patients and experts agree on the importance of SF and see it as one of the ultimate therapeutic goals in schizophrenia.^66–68^ As long-term SF depends on premorbid adjustment, childhood trauma, and genetic predisposition, preventive measures should target youth at high risk of developing schizophrenia as early as possible. High-risk profile groups should be identified at the intake and be provided with complex management plan by considering or targeting clinical and non-clinical factors. We found that supportive environment after psychosis onset also plays an important role in SF. Thus, if needed, clinicians should pay attention to provision of social support, occupational and social activities, involve and support patients’ families. The ethnicity and immigration status of patients should be well considered as being non-native to the area of living might bring extra burden.

### Future Research

Recent studies also highlight the importance of specific cognitive and negative symptom domains that predict or influence SF such as social cognition, social amotivation, and expressive deficit.^69,70^ Along with clinical disease aspects, environmental factors should be well considered and further investigated. Thus, fine-grained factors within the broad constructs of social support and family environment should be identified as they are potentially modifiable and can be used as an intervention target. Future studies could focus on social inclusion as a more independent measure from the disease but a key aspect of people’s lives.^71,72^ Additionally, predictors’ selection based on univariable analysis might result in exclusion of important predictors and is not advisable to be used as the main tool to decide on predictors potential importance.

### Strengths and Limitations

The strength of this study lies on its large and longitudinal design enriched with extended clinical, social, environmental, and functional measurements. As we lined up a series of complementary statical modeling to test for the associations, build and internally validate the prediction models, obtained estimates can be generalizable. However, we should acknowledge several limitations. Dropout was substantial, and given the demanding study procedures, patients with a severe symptom state or course were underrepresented. SF was not measured at the baseline and could not be used in the analysis while baseline SF could be highly predictive of the SF at follow-up. Clinical trajectories were identified based on measurements only partially preceding the outcome assessment; hence, the prediction by these trajectories should be interpreted cautiously. Finally, our prediction model was only internally validated.

## Conclusion

We identified patients with more severe trajectories of premorbid adjustment, cognitive deficits, and symptoms that showed lower long-term SF. We found that sex, ethnicity, polygenic risk score, childhood trauma, age, education, overall disease severity, cannabis use, urbanicity level, employment, relationship status, and unmet needs of social support were predictive of long-term SF. Additionally, intrafamilial factors predicted SF. We built and internally validated two separate models for a shorter (3-year) and a longer (6-year) follow-up and could explain up to 30% of the SF variation. Our model should be enriched by other factors which may improve its predictive accuracy in independent larger studies.

## Supplementary Material

sbad046_suppl_Supplementary_Materials
