# Supplementary material for: Understanding Lifelong Factors and Prediction Models of Social Functioning After Psychosis Onset Using the Large-Scale GROUP Cohort Study
Source: Schizophr Bull. 2023 Apr 27;49(6):1447–59. doi: 10.1093/schbul/sbad046 (PMC10686366; doi:10.1093/schbul/sbad046)
Supplement: sbad046_suppl_Supplementary_Materials [file sbad046_suppl_supplementary_materials.zip › Supplementary_Methods and Results_SF_Tiles-Sar.docx]

**Understanding lifelong factors and prediction models of social functioning after psychosis onset using the large-scale GROUP cohort study.**

Tiles-Sar *et.al*. 2022

**Table of Contents**

[Methods 1](#_Toc121190629)

[Missing values and drop-out 1](#_Toc121190630)

[Power calculation 1](#_Toc121190631)

[Statistical modelling 1](#_Toc121190632)

[Cross-validation 2](#_Toc121190633)

[Results 2](#_Toc121190634)

[Premorbid adjustment trajectories 2](#_Toc121190635)

[Premorbid adjustment, cognitive deficits, symptoms trajectories and SF 2](#_Toc121190636)

[Prediction models of SF 2](#_Toc121190637)

[Cross-validation 3](#_Toc121190638)

[References 5](#_Toc121190639)

## Methods

Missing values and drop-out

We compared patients who completed the follow-up assessments with drop-out patients (Supplementary Table S2). Since relationships between missing patterns and observed variables were found, we could, in approximation, confirm the missing at random (MAR) assumption for missing values. Among single imputation methods, Bayesian Stochastic regression imputation can be considered one of the best since it accounts for uncertainty by 1) adding error variance to the predicted values and 2) taking into account the uncertainty in estimating the regression coefficients of the imputation model. We additionally analyzed the observed dataset with maximum likelihood estimation and robust standard errors to account for missingness (reported in Supplementary materials).

Power calculation

Previous evidence showed that a sample size of 500 people is sufficient for obtaining effect estimates close to the true population values.^1^ We used the sjstats package in R to perform a power analysis.^2–4^As 35 clusters (the maximum number of categories in the final multivariable model) were included in a two-level model design and assuming a small effect size (Cohen’s d = 0.30), at least 668 cases were needed to gain a power of 0.80 at a p-value of 0.05. The imputed dataset hence contained sufficient observations.

Statistical modelling

Within group-based trajectory modeling, the missing values were handled by fitting the model using the maximum likelihood estimation. The main criteria for the model fit were the Bayesian information criteria (BIC) and logged Bayes factor (2*ΔBIC=2*(BIC_more_complex_model- BIC_less_complex_model)). Model accuracy and entropy were defined at the cut-off above 0.70. First, we did the stepwise model fit comparison by varying number of trajectories from 1 to 10 trajectories (subgroups) assuming the maximum quadratic slope and no drop-out model (Supplementary Table S5). Next, the best-fitting model was calibrated with respect to the maximum polynomial order for each trajectory as showed in Supplementary Table S6. Thus, quadratic slope was dropped in favor of linear shape if P>0.5 for a given trajectory order.

On the stage of prediction modelling, inclusion of predictors was based on univariable analysis, p-value < 0.25. Step-wise predictors' exclusion was based on collinearity measure - variance inflation factor (VIF) above 2 in the full model. Further, we trained the model on the whole dataset with backward predictor selection. The final best-fitted model was validated on 1000 full-size bootstrap samples. The averaged estimates based on these samples were automatically calculated. The bias was calculated as the difference between averaged bootstrap estimates and previously obtained estimates on the imputed dataset. Confidence interval (95%) based on bootstrap SE was calculated.

Cross-validation

Accuracy estimates were acquired with the leave-one-out cross-validation algorithm run on the full (models with all predictors) and final best-fitted prediction models ({lvmisc} package in R).^5^ We reported mean absolute error (MAE), mean absolute percent error (MAPE), and root mean square error (RMSE) for the models without and with cross-validation. Cross-validated accuracy estimates should be considered more accurate.

## Results

Premorbid adjustment trajectories

The best model was chosen based on the most optimal combination of the logged Bayes factor, an averaged posterior probability, and the number of patients per group. Identified groups vary in initial severity level (i.e., intercept), shape (i.e., linear and quadratic), and speed of decline (i.e., the slope). Thus, group 2 and 3 had a quadratic shape and the most rapid decline (slope of 0.02±0.01, p-value<0.01 and -0.02±0.01, p-value=0.05 respectively), while groups 1 (0.09±0.01, p-value<0.01), 4 (0.10±0.01, p-value<0.01), 5 (0.04±0.01, p-value<0.01) and 6 (0.11±0.02, p-value<0.01) had a linear shape with modest slopes. Group 5 had the smallest slope and remained stable over time.

Premorbid adjustment, cognitive deficits, symptoms trajectories and SF

In comparison to the null model (no fixed effect is included), the models with trajectories performed significantly better. Thus, the model for 3-year follow-up showed significant drop in AIC (8207.4 versus 8046.4), BIC (8227.5 versus 8106.7), and logLik (-4099.7 versus -4011.2) with chisq=177, and p-value<2.2e-16. The model for 6-year follow-up also showed great improvement in comparison to the null model as AIC decreased from 8123.2 to 7948.5, BIC from 8143.3 to 8008.8, lokLik from -4057.6 to 3962.3 with chisq=190.68, and p-value<2.2e-16.

Prediction models of SF

The final models presented a better fit according to AIC at second wave 7352.80 versus 7359.30 (chisq=13.49; p-value=0.19) and at third wave 7297.60 versus 7304.80 (chisq=6.76; p-value=0.45) and BIC at second wave 7476.60 versus 7532.60 and at third wave 7436.30 versus 7478.20. Based on the distribution of the residuals, both assumptions of normality and homoscedasticity were met. Specifically for polygenic risk score, we calculated model performance for SF at 3-year follow-up with (marginal R^2^ =0.28, conditional R^2^=0.29) and without (marginal R^2^ =0.27, conditional R^2^=0.30) this predictor. Validation analysis confirmed the observed effect for most predictors, with less than 1% of bias in effect estimation. Bias for the models' performance was 5% of the estimated performance in the imputed dataset. The explained variance by fixed effect only (marginal R2) was 0.27 [95% CI:0.23, 0.30] for the second wave and 0.26 [0.22, 0.31] for the third wave.

Cross-validation

The results of cross-validation are presented below in the Table 1. The difference between validated and non-validated models’ performance was minor for the 3-year follow-up, although higher for the models of 6-year follow-up. Across validated models, performance is very similar for 3-year and 6-year follow-ups. We earlier observed similar marginal (attributed to fixed effect only) pseudo-R^2^ in the models for both follow-ups, but higher conditional (attributed to fixed and random effects) pseudo-R^2^ for 6-year follow-up. Possibly, the random effect was diminished by leave-one-out cross-validation which resulted in similar performance for both follow-ups. In validated and non-validated models, final models with reduced set of predictors performed better than full models with all predictors.

| **Table 1.** The model fit characteristics estimated by MAE, MAPE, RMSE across the prediction models of SF at 3-year and 6-year follow-ups. | | | |
| --- | --- | --- | --- |
| Model | MAE | MAPE | RMSE |
| **Prediction models of 3-year follow-up** | | | |
| **Not validated** **full** prediction model of SF at 3-year follow-up | 6.25 | 5.68% | 7.89 |
| **Validated full** prediction model of SF at 3-year follow-up | 6.48 | 5.89% | 8.18 |
| **Not validated** **final best-fitted** prediction model of SF at 3-year follow-up | 6.29 | 5.71% | 7.95 |
| **Validated** **final best-fitted** prediction model of SF at 3-year follow-up | 6.45 | 5.86% | 8.14 |
| **Prediction models of 6-year follow-up** | | | |
| **Not validated full** prediction model of SF at 6-year follow-up | 3.38 | 3.04% | 4.19 |
| **Validated full** prediction model of SF at 6-year follow-up | 6.40 | 5.76% | 7.93 |
| **Not validated final best-fitted** prediction model of SF at 6-year follow-up | 3.37 | 3.03% | 4.16 |
| **Validated final best-fitted** prediction model of SF at 6-year follow-up | 6.38 | 5.74% | 7.88 |

## References

1. Loughran T, Nagin DS. Finite sample effects in group-based trajectory models. *Sociol Methods Res*. 2006;35(2):250-278. doi:10.1177/0049124106292292

2. sjstats package | R Documentation. https://www.rdocumentation.org/packages/sjstats/versions/0.17.5. Accessed May 7, 2020.

3. Snijders TAB. Power and Sample Size in Multilevel Linear Models. In: *Encyclopedia of Statistics in Behavioral Science*. Chichester, UK: John Wiley & Sons, Ltd; 2005. doi:10.1002/0470013192.bsa492

4. Core Development Team R. A Language and Environment for Statistical Computing. *R Found Stat Comput*. 2020.

5. Lucas Veras. lvmisc: Veras Miscellaneous. 2022.
